# Practical Notes and Formulæ

**Published:** 1880-02-20

**Authors:** 


### PRACTICAL NOTES AND FORMULÆ.

**Sulphate of Zinc in Anemia.**—Dr. Fenwick notes that in anæmia, coexistent as it often is with cardiac hypertrophy, iron is objectionable, and may be advantageously discarded for zinc sulphate, as in the following formula :

| | | |
|---|---|---|
| ℞ | Zinci sulphatis | |
| | Ext. gentianæ | aa gr. ij. |
| | Pill. rhei. comp | gr. j. |

For one pill, twice or thrice daily.—*Med. and Surg. Reporter.*

**Chloral in Diphtheria.**—

| | | | |
|---|---|---|---|
| ℞ | Chloral hydratis | ʒj. | |
| | Aquæ | ℥j. | M. |

Use as a gargle every hour or two.

Dr. R. Carney, in the Canada Lancet, says : "This is a specific in diphtheria; it has a wonderful effect, the patches rapidly peeling and the patient promptly improving." It may be applied by a sponge swab in infants.

**Coto Bark in Diarrhœa.**—I have tried coto bark with prompt success in a case of diarrhœa with tubercular complications. The following is the formula I have employed :

| | | |
|---|---|---|
| ℞ | Fl. ex. coto bark | |
| | Com. tr. cardamon | aa ℥ ij. |
| | Mucilage acacia | |
| | Syrup | aa ℥ ss |
| | Cinnamon water, q. s. add | ℥ viij. |

M. Dose, a tablespoonful every three hours.—Dr. J. B. Crandall in *Therap. Gazette.*

**Gastric Catarrh.**—

| | | |
|---|---|---|
| ℞ | Sulphate magnesia | ʒss: |
| | Bicarb. potash | grs xxx. |
| | Salicylic acid | grs. x. |
| | Water | ℥iv. |

To be taken after meals as soon as the symptoms of acidity are felt approaching.—*Ec. Med. Journal.*

**Dysmenorrhœa.**—A writer, in Brief, used the following remedy successfully to relieve the pain of dysmenorhœa :

| | | |
|---|---|---|
| ℞ | Camphor | 10 grains. |
| | Dover's powders | 10 grains. |
| | Ext. hyoscyamus | 10 grains. |
| | M. Ft. pill 10. | |

Dose, two pills every two hours.

**The Purity of Chloroform.**—It being very important that chloroform for anæsthetical purposes should be pure, the following simple tests are recommended by the author. If chloroform is dropped on paper and allowed to evaporate, the last portion on being inhaled has a characteristic pleasant smell, and leaves the paper perfectly dry and odorless; impure chloroform, however, possesses a disagreeable irritating odor, which it imparts to the paper.

Pure chloroform does not redden blue litmus or give even a cloudiness with silver nitrate. If it should do either, it contains hydrochloric acid or products of decomposition of some other chlorides. Pure chloroform remains perfectly colorless when boiled with potassa; the presence of aldehyde causes a brown coloration.

When shaken with concentrated sulphuric acid and allowed to stand for half an hour, the two liquids should separate into two colorless layers. The presence of alcoholic chlorides produces a brown coloration.—*New Remedies.*

**Mithie's Elixir.**—This is a carminative elixir, which is prepared according to the following formula :

| | | |
|---|---|---|
| ℞ | Ginger | ℥ ½ |
| | Cinnamon | ʒ 2 |
| | Long pepper | ʒ 2 |
| | Galanga | ʒ 1 |
| | Nutmeg | ʒ 1 |
| | Cloves | ʒ 1 |
| | Cardamom | ℈ 1 |
| | Alcohol | fl ℥ 6 |

Digest for 14 days and filter. Dose, ¼ teaspoonful in some suitable vehicle.

**Soda Benzoate.**—Dr. Wm. B. Davis, in Cincinnati Lancet and Clinic, reported having had good success in the use of the soda benzoate in a case of ulcerated sore throat. There was marked improvement in twenty-four hours after commencing its use. In a severe case of scarlet fever with ulcerated sore throat there was evident improvement at first, but the patient had succumbed to the poison that day. His formula was for adults :

| | | |
|---|---|---|
| ℞ | Sodii Benzoat | |
| | Aqua menth. pip | aa ℥ ss. |
| | Syr. bal. Tolu | ℥ ii. |

M. fl. ʒss every three hours.

For children :

| | | |
|---|---|---|
| ℞ | Sodii benzoat | ʒ i. |
| | Aq. menth. pip | ℥ ii. |
| | Syr. bal. Tolu | ℥ i. |

M. fl. ℥i every two hours.

**Hagan's Magnolia Balm.**—This balm is said by Chandler to be a colorless fluid containing 23.07 of zinc oxide. The bottle contains 3½ fl. oz. of liquid, containing 2 drachms of glycerin and 262 grains of zinc carbonate, together with a trace of carmine, and flavored with lavender and bergamot.—*New Remedies.*

**Anti-odontalgic.**—The following is from Dr. J. Pirnat, in the Medical and Surgical Reporter.

"The following toothache panacea I have used for the last ten years, viz. :

℞ Chloral hydratis..............................<br>

Gum camphoræ..............................aa ʒj.<br>

Olei caryophilli.............................. gtts.xx

Sig. Saturate a small piece of cotton and plug the cavity with it."

**Constipation.**—Dr. Griswold, in Medical Brief, says : In the constipation of pregnancy, or constipation resulting from a torpid condition of the bowels from any cause, also in those chronic dyspepsias, when acidity is very prominent, and action of the bowels is seldom obtained except by artificial means, the following will be found of very great benefit. It has about it an air of old-fashionedness, but its worth is a matter of fact.

℞ Fol. Senna.............................. ʒiv.<br>

Manna.............................. ʒiv.<br>

Pulv. Angustura bark.............................. ʒij.<br>

Pulv. gentian.............................. ʒi.<br>

Pulv. rhei.............................. ʒ1½.<br>

Sem. annis.............................. ʒ1½.<br>

Capsicum.............................. ʒ½.<br>

M. et add<br>

Boiling water.............................. 1 pint.<br>

Spts. turpentine.............................. ℥vi.<br>

Steep one hour, strain and bottle when cool.

Dose, one tablespoonful morning and night.—*Brief.*

**Alcott's Red Drops.**—The following is the formula for the celebrated Alcott's Red Drops, which formerly enjoyed a great reputation in this section, in the cure of obstinate diarrhœa, and which I find as beneficial now as it probably was forty years ago.

℞ Pulv. rhei.............................. ʒj.<br>

Sach. alba.............................. ℥iv.<br>

Pimento.............................. ℥½.<br>

Boiling water.............................. 1 pint.<br>

M. and when cool add<br>

Tinct. opii.............................. ℥1½.<br>

Sprts. ammo. ar.............................. ʒv.<br>

Sprts. vini gallici.............................. ℥ij.

Dose, one to two teaspoonfuls every two or three hours.—*Brief.*

**Netolitzky's Bromine Inhalation.**—

℞ Bromine.............................. grs. 8 to 16.<br>

Bromide of potassium.............................. grs. 8 to 16.<br>

Distilled water..............................fl ℥5 to 7.

Dissolve. To be poured, in small quantities at a time, upon a sponge or upon lint, for inhalation in croup. The effects of this solution are very favorably spoken of.—*Prag. Med. Wochensch.*

**Purgative in Piles.**—Dr. Barker's prescription is as follows:

| | | |
|---|---|---|
| ℞ | Pulv. aloes soc | 20 grains. |
| | Sapo cast | 20 grains. |
| | Ext. hyosciami | ʒ ½. |
| | Pulv. ipecac | 5 grains. |
| | M. Ft. pil. No. 20. | |

Dose, one pill morning and evening.

If the rectum is irritable, with small, thin, frequent evacuations, he substitutes opium for the hyoscyamus and uses less aloes.—*Brief.*

**For Acute Rheumatism.**—Solution of salicylic acid, particularly useful in acute rheumatism.

| | | |
|---|---|---|
| ℞ | Acidi salicylici | ʒj |
| | Sodii biboratis | ʒ ½. |
| | Glycerine | ʒ ij. |
| M. | Heat until dissolved, and add | |
| | Glycerine | ℥ j. |
| | Aqua q. s. ut ft | ℥ ij. |

Dose, one teaspoonful every three hours.

**A Good Blacking.**—Probably the best ordinary blacking which can be produced is made by the following formula, which has been long in use.

Mix intimately .

| | |
|---|---|
| Molasses | 1℔ |
| Best bone black in very fine powder | 1℔ |
| Olive oil | ¼℔ |
| Sulph. acid previously diluted with ¾℔ of water | ¼℔ |

The whole is allowed to stand for 3 hours or longer, and afterwards as much water is added as is necessary to give it the proper consistence. —*New Remedies.*

**Remedy for Corns.**—Mr. Gezow, a Russian apothecary, recommends the following as a sure remedy for corns, stating that it proves effective within a short time, and without causing any pain.

| | | |
|---|---|---|
| ℞ | Salicylic acid | 30 parts. |
| | Extract of cannabis indica | 5 parts. |
| | Collodion | 240 parts. |

To be applied by means of a camel's-hair pencil.—*Pharm. Zeit. F. Russl.*

**Palatable Epsom Salt.**—M. Yvon states that the disagreeable taste of sulphate of magnesia may be completely concealed by the addition of a few drops of the essence of mint, provided that the quantity of the vehicle be small. He advises that ʒ vi. of the sulphate should be dissolved in about ℥i. of water, two or three drops of the essence of mint being then added; or the flavoring agent may be added to the salt, and the patient directed to dissolve the whole in as small a quantity of water as possible.—*New Remedies.*

